# Mucosa-associated lymphoid tissue in individuals with AIDS

**DOI:** 10.1590/S1808-86942011000300007

**Published:** 2015-10-19

**Authors:** Janainna Grazielle Pacheco Olegario, Renata Calciolari Rossi e Silva, Vicente de Paula Antunes Teixeira, Eumênia Costa da Cunha Castro, Rosana Rosa Miranda Corrêa

**Affiliations:** 1 Master's degreee, doctoral student, Triangulo Mineiro Federal University (Universidade Federal do Triângulo Mineiro - UFTM); 2 Doctoral degree. Professora at the Oeste Paulista University (Universidade do Oeste Paulista - UNOESTE); 3 Doctoral degree. Full professor, general pathology discipline, Triangulo Mineiro Federal University - UFTM; 4 Doctoral degree. Professor at the Triangulo Mineiro Federal University - UFTM; 5 Doctoral degree. Adjunct professor, general pathology discipline, Triangulo Mineiro Federal University - UFTM; General pathology discipline, Triangulo Mineiro Federal University, Uberaba, Minas Gerais, Brazil

**Keywords:** acquired immunodeficiency syndrome, fibrosis, immunoglobulins, lymphoid tissue

## Abstract

Vestibular folds (VF) protect upper airways, but contain fewer immune cells in AIDS patients, which affects the structure of lymphoid follicles (LF).

**Objective:**

To characterize fibrosis and immunoglobulin production in vestibular fold lymphoid tissues of AIDS patients with or with no infection and malnutrition.

**Materials and Methods:**

A retrospective study of 71 adult vestibular fold autopsy specimens. The morphological analysis was done using the picrosirius staining method. Immunohistochemical methods consisted of anti-IgA, anti IgG, and anti IgM antibodies.

**Results:**

Fibrosis was less intense in AIDS patients compared to subjects without AIDS; the same applied to patients with infection or malnutrition. IgA and IgG titers were higher in AIDS patients; IgM titers were higher in cases with infection.

**Conclusion:**

This study helps understand variations in lymphoid follicle components of AIDS patients; it also shows the influence of architectural changes and the effect of associated respiratory infection and malnutrition on lymphoid follicle function.

## INTRODUCTION

AIDS (the Acquired Immunodeficiency Syndrome), identified in 1981, arose as an epidemic caused by the human immunodeficiency virus,^1^ which could result in decreased and dysfunctional CD4 T lymphocytes. Other immune cells could harbor the virus, and thus lymphoid tissues, such as those in the larynx, were perceived as possible viral replication structures.^2^, ^3^, ^4^, ^5^

Lymphoid follicles result from the union of different types of B and T lymphocytes; when close to mucosae, they produce substances that active the local immune response against infectious agents. Lymphoid follicles located in vestibular folds (two thick sagittally oriented laminae, with a double-sized mucosa, that arises within the supraglottic wall)^6^ protect upper airways, similarly to lymphoid tissues associated with mucosae.^7^, ^8^, ^9^

Mucosa-associated lymphoid tissues prevent the penetration of many microorganisms into the body.^10^, ^11^, ^12^ Cytokines produced in lymphoid follicles cause plasmacytic differentiation and antibody production (mostly IgA in the respiratory tract), a component of mucosal cell secretions, including that of the vestibular folds.^5^, ^13^ Cell-mediated and humoral immunity are unable to control infection in HIV-positive subjects, resulting in loss of several lymphocyte functions and increased susceptibility to secondary and opportunistic infection. Part of the immune dysfunction is in polyclonal B lymphocytes; these are constantly activated, which may result in spontaneous hypergammaglobulinemia.^14^, ^15^ Other signs of B lymphocyte abnormalities are a high rate of tumors originating from these cells in HIV-positive patients, and poor regulation of the expression of several surface molecules.^16^ It has been shown that serum IgA levels tend to increase as a results of HIV infection, a phenomenon that may predict the progression of AIDS. Although changes in IgA secretion may be expected following HIV infection, little is known about the humoral responses in mucosae in the presence of immunodeficiency.^15^

Respiratory infection is one of the main causes of mortality and morbidity in AIDS patients.^17^, ^18^, ^19^, ^20^ Lymphoid tissues in IgA-secreting mucosa are the main local protective mechanism for the respiratory tract; activation of these tissues is the goal of vaccines aiming at conferring immunity in upper airways to prevent respiratory infection.^10^, ^11^, ^13^

Aside from changes in IgA secretion, a few authors have suggested that increased deposition of collagen in AIDS patients may also deplete CD4 T cells. Increased collagen neoformation is associated with chronic activation of the immune system; there is a relationship between collagen neoformation and loss of the lymphoid tissue structure.^21^, ^22^, ^23^, ^24^

Studies done in our department have demonstrated the nature of cells composing the lymphoid follicles in vestibular folds of adults^9^ and their depletion in AIDS cases.^5^ Because of the importance of the immune system for local protection of mucosae, knowing the composition, arrangement and function of lymphoid follicles in vestibular folds is the first step in understanding the mechanisms involved in respiratory infection in AIDS patients. Thus, the purpose of this study was to assess the effect of fibrosis on the architecture of lymphoid follicles in the vestibular folds of autopsied AIDS adults, and to assess their function by quantifying the immunoglobulins produced in these follicles.

## MATERIALS AND METHODS

This paper describes a retrospective cross-sectional study of 290 autopsies done from 1993 to 2007. Information such as age, sex, weight, and cause of death were recorded from autopsy reports. Excluded cases were those in which the larynx was not studied, or that were aged under 18 years, or cases without AIDS that could not be paired by age with the AIDS group. This resulted in 71 vestibular folds of autopsied adults, of which 52 were AIDS cases and 19 had no AIDS. In subjects aged 60 years or more, a body mass index (BMI) below 22.0 kg/m^2^ was considered undernourished, and a BMI over 27.0 kg/m^2^ was considered overweight. In subjects aged less than 60 years, a BMI below 18.5 kg/m^2^ was undernourished and a BMI over 25.0 kg/m^2^ was overweight.^25^, ^26^

The institutional review board approved this study on 30 May 2004 (no. 481).

Vestibular fold specimens were included in paraffin and 4 μm sections were made. Immunohistochemical studies were made for a later morphometric analysis of cells in the lymphoid follicles of vestibular folds and immunoglobulin expression. Tripsin was used in the antigen recovery process. Slides were washed with a PBS 0.05M + Triton X-100 0.05% buffer. All antibodies were incubated during a mean 2-hour period. The LSAB complex (DAKO®) was added and left over the sections during 30 minutes. Next, the material was incubated with 3,3'-Diaminobenzidine (DAB) at room temperature. The slides were then washed with distilled water, counterstained with hematoxylin and mounted in entellan. For the morphometric analysis of false vocal fold lymphoid follicle cells, immunohistochemistry was done for B and T lymphocytes, macrophages, and follicular dendritic cells; primary anti-B-cells (Biogenex, 1:100), anti-CD3 (Dako, 1:40), anti-CD68 (Dako, 1:80), and anti-follicular dendritic cells (Dako, 1:80)^5^ antibodies were used. The following antibodies were used for evaluating the immunoglobulins: anti-IgA (Novocastra®, 1:1000), Anti-IgG (Novocastra®, 1:1000), and anti-IgM (Novocastra®, 1:800). Expression of each immune marker was quantified throughout the histological section using the KS 300 software (Kontron-Zeiss®).

Slides were picrosirius stained for the analysis of fibrosis (a saturated aqueous solution of picric acid with 0.1g% Sirius red F3b - Sirius red F3B-Bayer®) with a hematoxylin counterstain. Collagen was quantified throughout the vestibular fold section under polarized light at 200X magnification. Results were expressed as the percentage of collagen per field, using the KS 300 software (Kontron-Zeiss®).

An electronic Microsoft Excel spreadsheet and the SigmaStat® version 2.0 software were used for the statistical analysis. Statistically significant differences were those where the probability of rejecting the null hypothesis was less than 5% (p<0.05).

## RESULTS

**Figure 1:**
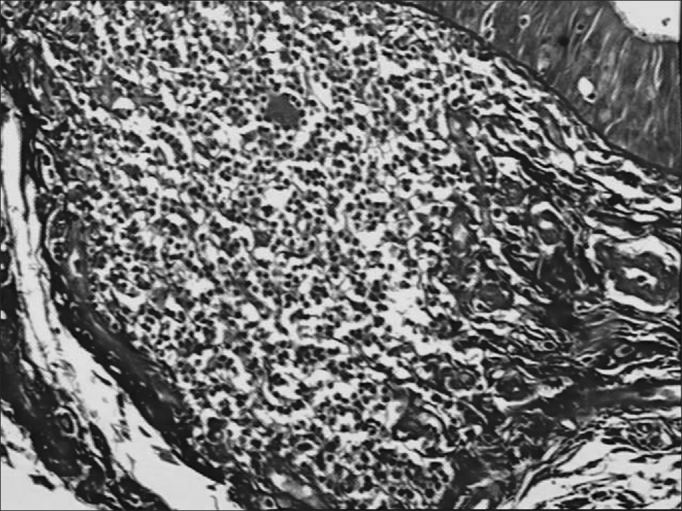
Picro-sirius in FVF of an AIDS patient (PS 200X).

**Figure 2:**
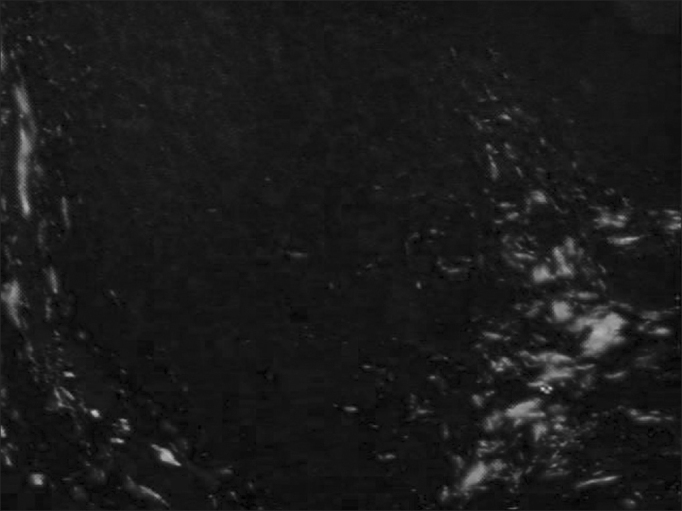
Picro-sirius in FVF of an AIDS patient under polarized light (PS 200X).

Of 71 subjects in the sample, 52 (73.24%) were diagnosed with AIDS; the mean age was 37.5 years, ranging from 21 to 61 years. There were 19 AIDS-free subjects (26.8%), where the mean age was 41.0 years, ranging from 23 to 62 years. The morphometric analysis of vestibular fold lymphoid follicles showed that fibrosis was less intense in AIDS patients compared to subjects without AIDS (p=0.002) (Figs. 1 and 2). Cases with AIDS and with respiratory tract infection had less fibrosis. There was significantly less fibrosis in undernourished AIDS cases compared to normally nourished AIDS cases (p ≤ 0.001) (Table 1).

**Table 1:** Comparison of the amount of fibrosis relative to the diagnosis of AIDS, respiratory infection, and undernourishment in autopsied subjects.

Fibrosis n(%) Median (Minimum-Maximum)			
With AIDS*	52 (73.2)	3.5 (0.5-13.3)	T=474599.000; *p*=0,002
Without AIDS*	19 (26.8)	4.3 (0.9-11.2)	
With Infection/AIDS*	38 (73.1)	3.1 (0.5-12.6)	T=253822.500; *p*≤0,001
No Infection/AIDS*	14 (26.9)	5.2 (0.9-13.3)	
Undernourished/AIDS*	23 (45.1)	4.5 (1.2-12.6)	T=412033.000; *p*≤0,001
Normally nourished/AIDS*	28 (54.9)	12.8 (0.5-13.3)	

* raised to the third power and expressed as μm^2^.

**Figure 3:**
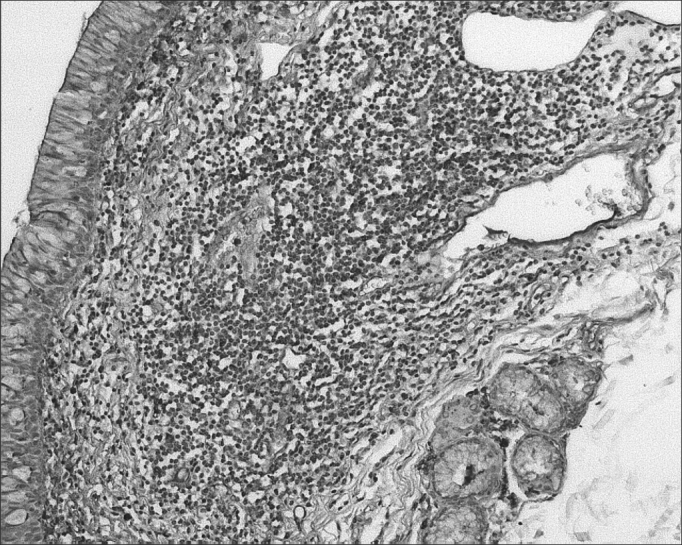
Immunohistochemistry for IgA in FVF of an AIDS-free patient (PAP 200X).

**Figure 4:**
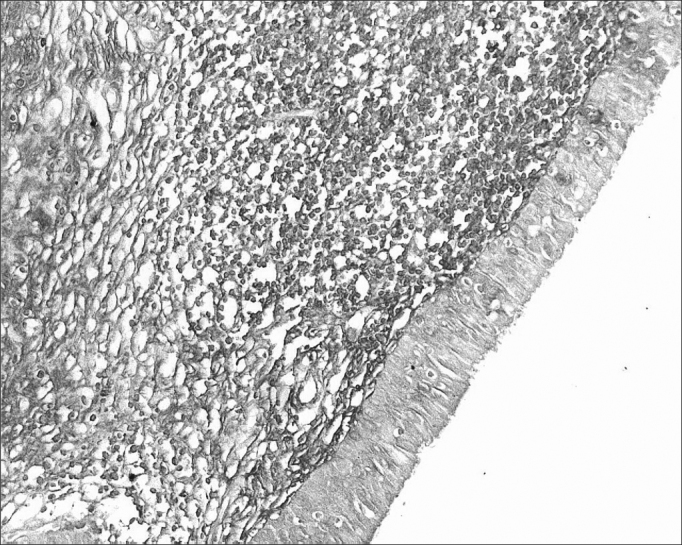
Immunohistochemistry for IgA in FVF of an AIDS patient (PAP 200X).

**Figure 5:**
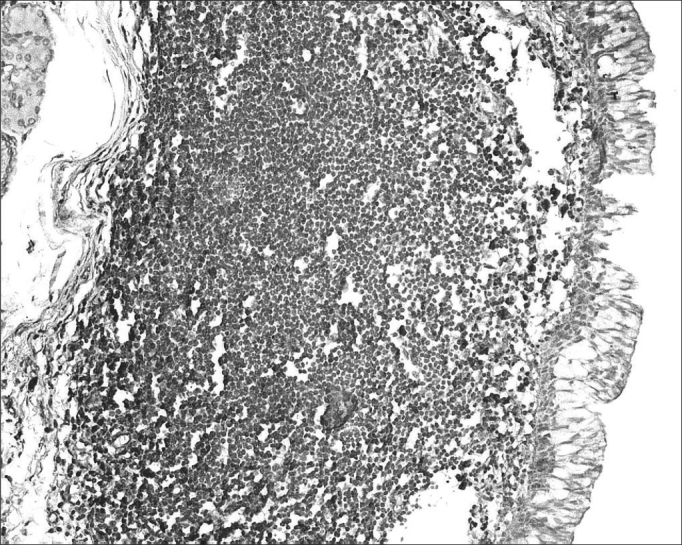
Immunohistochemistry for IgG in FVF of an AIDS-free patient (PAP 200X).

**Figure 6:**
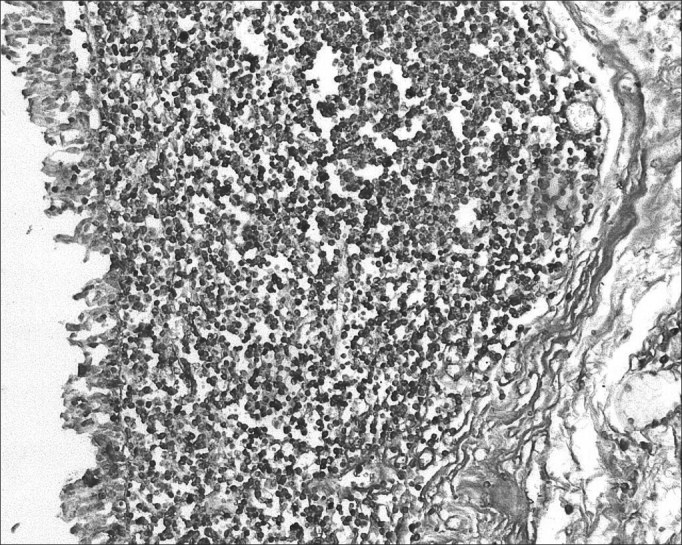
Immunohistochemistry for IgG in FVF of an AIDS patient (PAP 200X).

**Figure 7:**
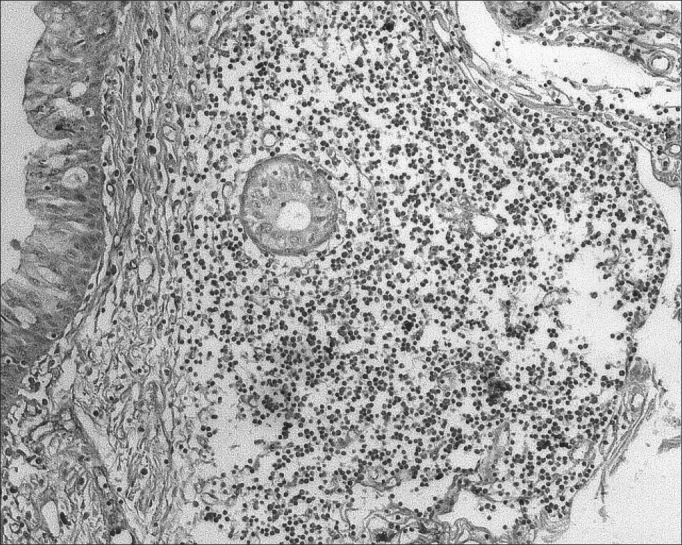
Immunohistochemistry for IgM in FVF of an AIDS-free patient (PAP 200X).

**Figure 8:**
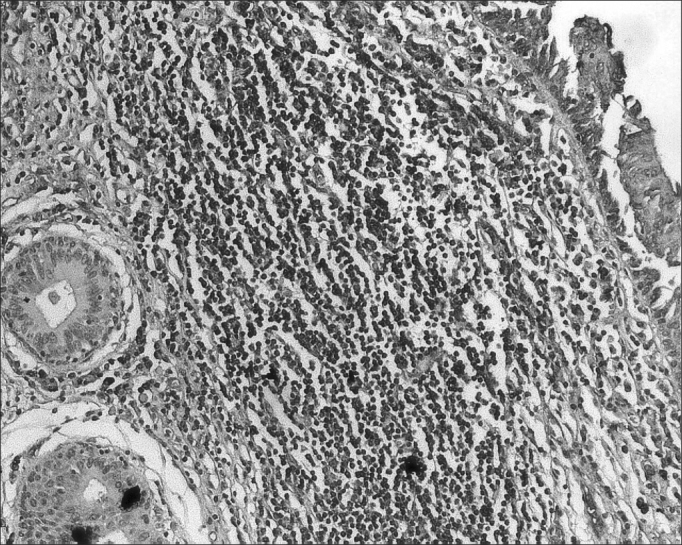
Immunohistochemistry for IgM in FVF of an AIDS patient (PAP 200X).

The morphometric analysis of immunoglobulins showed that subjects with AIDS had significantly lower levels of IgM (Figs. 7 and 8), and significantly higher levels of IgG (Figs. 5 and 6) and IgA (Figs. 3 and 4). Cases with aids and respiratory tract infection had significantly higher levels of IgM; undernourished cases had significantly higher levels of IgA and significantly lower levels of IgM (Table 2).

**Table 2:** Comparison of the quantity of immunoglobulins relative to the diagnosis of AIDS, respiratory infection, and undernourishment in subjects.

Immunoglobulins Median (Minimum-Maximum)				
	n (%)	IgA*	IgG*	IgM*
With AIDS*	52 (73.2)	84.9 (2.4 - 1165.8)	136.3 (0.7 - 744.1)	28.3 (0.3 - 1020.5)
Without AIDS*	19 (26.8)	76.6 (9.6 - 1018.66)	82.9 (0.8 - 718.3)	44.9 (0.2 - 621.1)
		T = 127837.500; p=0.019	T = 99835.000; p≤0.001	T = 97021.500; p≤0.001
With Infection/AIDS*	38 (73.1)	87.6 (2.4 - 1165.8)	120.9 (0.7 - 726.3)	28.9 (0.3 - 1020.5)
No Infection/AIDS*	14 (26.9)	86.3 (2.9 - 940.2)	148.1 (6.6 - 744.1)	28.1 (3.6 - 294.2)
		T = 90316.000; p=0.720	T = 79062.000; p=0.237	T = 63961.000; p=0.043
Undernourished/AIDS*	23 (45.1)	101.0 (2.7 - 1126.9)	139.7 (3.5 - 744.1)	21.5 (0.9 - 1020.5)
Normally nourished/AIDS*	28(54.9)	77.6 (2.4 - 1165.8)	120.0 (0.7 - 700.6)	31.7 (0.3 - 572.3)
		T = 143291.000; p≤0.001	T = 121616.000; p=0.562	T = 78309.000; p≤0.001

* raised to the third power and expressed as μm^2^.

The following correlations were found: negative between fibrosis and the number of T lymphocytes (rS=-0.213; p=0.0657), B lymphocytes (sR=-0.0500; p=0.685), and follicular dendritic cells (sR=-0.0275; p=0.819); positive between fibrosis and the number of macrophages (sR=0.0980; p=0.415); negative and significant between fibrosis and the percentage of IGA (sR=-0.0818; p=0.00762), and positive and significant between fibrosis and the percentage of IgG (sR=0.0874; p=0.00644) and IgM (sR= 0.108; p=0.00194).

## DISCUSSION

Our study showed that fibrosis was less intense in AIDS patients, and was negatively related with the percentage of T lymphocytes. In HIV patients, collagen neoformation and fibrosis contribute to CD4^+^ T cell depletion and limit recovery of the immune response. An increased predisposition to collagen neoformation may be related with a decreased virgin CD4^+^ T lymphocyte cell population, in which the phenotype is not activated before antiretroviral therapy. Fibrosis may be one of the causes of failure in immune recovery during treatment in spite of a suppressed viral replication.^21^, ^22^, ^23^, ^24^ These data agree with our findings, in which the amount of fibrosis was negatively correlated with the number of T lymphocytes; although fibrosis was less intense in AIDS patients, it was mostly present in the parafollicular zone, taking up the place of T lymphocytes. Such a change in the parafollicular zone may alter the general structure of lymphoid follicles, thereby decreasing the number of other cell types. Although there were more macrophages relative to fibrosis, we believe that these cells are generally involved in tissue repair, and therefore with collagen production,^27^ which explains the positive correlation we encountered.

Fibrosis was less intense in AIDS patients that also presented respiratory infection and malnutrition. Respiratory infection is considered the main cause of death in AIDS patients;^19^, ^20^, ^24^ it occurs in later stages of the disease, when an altered nutritional status is also found.^28^, ^29^, ^30^ A study of SIV-infected monkeys (with the simian immunodeficiency virus), in which there is a similar infection to that of humans, demonstrated increased fibrosis in lymphoid tissues. These animals, however, were evaluated during a short period following infection; there was no report about chronic SIV infection,^24^ which could be similar to severe AIDS cases as we found among our sample - which culminate in complications (infection and undernourishment) and death.

Although subjects were not evaluated as to the use of antiretroviral therapy, other studies have shown that if treatment is started early during the symptomatic phase of the disease, it may help balance helper and regulator T cells, which in consequence results in less collagen neoformation^24^, ^31^ - as evidenced in our cases - and may explain the amount of fibrosis in autopsied AIDS subjects.

There was less IgM and more IgA and IgG in vestibular fold lymphoid follicles of AIDS cases. Other studies have shown that IgA secretion is decreased in the parotid and palatine tonsils, suggesting that systemic and mucosal immune responses vary in HIV infection.^14^, ^15^, ^32^ In this study, secretory IgA levels are markedly reduced; such a difference between the systemic and mucosal response could contribute to/or explain the reduction in mucosal defenses, which results in opportunistic infections. Secreted IgG concentrations were elevated in HIV patients, which could reflect serious alterations in the mucosal barrier. Activated polyclonal B lymphocytes and IgG secretors could explain these increased concentrations. A significant increase in serum IgA could be a result of polyclonal lymphocyte activation, which produce more monomeric IgA (IgA1) and break down mucosal secretory IgA.^14^, ^15^, ^32^

The immunoglobulin titers, especially IgM, were higher in AIDS patients with respiratory infection. Several studies have shown that immunoglobulin serum levels tend to increase because of HIV - polyclonal B lymphocytes are activated, which may be predictive for the progression of AIDS. Although the role of serum IgA in immunity remains unclear, evidence suggests that secretory IgA has a fundamental function in mediating immunity against infection of mucosal surfaces. However, little is known about humoral responses in the presence of immune defficiencies.^14^, ^15^ This study is the first to evaluate the expression of different secretory immunoglobulins in AIDS patients; it shows that increased levels of these immunoglobulins may be an attempt by the immune system in the mucosa to respond to infection.

In our cases, infected subjects died because of opportunistic infection; IgM was increased compared to other immunoglobulins. IgM is an important link between innate and specific immune responses; it can retain and increased the immunogenicity of pathogens early in infection, resulting in rapid neutralization. It also promotes agglutination of several microorganisms, stopping them from disseminating and reducing the viral/microbial titers in the body. IgM also causes IgG responses to mature, and activates the complement system in immune responses.^33^ Thus, we believe that the death of patients took place early in infection, which explains the IgM titers we found.

IgA and IgG levels were higher and IgM levels were lower in undernourished AIDS patients. Protein synthesis is compromised in undernourished patients, which also decreases the amount of collagen, as shown before. An altered metabolism, hormone unbalances, and high concentrations of cytokines have been documented in late phases of infection.^30^ Thus, the time elapsed for undernourishment to develop may be associated with prolonged cases of AIDS, which explains the unbalanced production of immunoglobulins and increased expression of IgA and IgG - which are generally higher in chronic conditions.

Fibrosis was negatively related with IgA and positively related with IgG and IgM. The structure of lymphoid tissues is important for its homeostasis; more specifically, the structure of secondary lymphoid tissues is essential with its supportive function for the homeostasis of the immune system as a whole. Collagen deposition in these tissues limit the resident cell population and alters the follicular structure.^21^, ^22^ Thus, fibrosis changes the constituents and the architecture of lymphoid follicles, altering their function as observed in the decreased secretion of IgA, its main immunoglobulin, in AIDS.

## CONCLUSION

This study helps understand the variation of vestibular fold lymphoid follicles constituents in autopsied AIDS patients, as well as the effect of changes in architecture, associations with other conditions - such as respiratory infection and undernourishment - on the function of lymphoid follicles, especially in severe AIDS cases that led to death. This study also provides information for understanding in greater depth the mechanisms of the virus in specific areas of the body, such as the upper respiratory tract, showing how these anatomical structures may react to the virus and the secondary complications and opportunistic diseases associated with AIDS.
